# Neurobiological differences in mental rotation and instrument interpretation in airline pilots

**DOI:** 10.1038/srep28104

**Published:** 2016-06-21

**Authors:** Ronald Sladky, Irene Stepniczka, Edzard Boland, Martin Tik, Claus Lamm, André Hoffmann, Jan-Philipp Buch, Dominik Niedermeier, Joris Field, Christian Windischberger

**Affiliations:** 1 MR Center of Excellence, Center for Medical Physics and Biomedical Engineering, Medical University of Vienna, Austria; 2 Cognitive Science Research Platform, University of Vienna, Austria; 3 National Aerospace Centre (NLR), Amsterdam, Netherlands; 4 Social, Cognitive and Affective Neuroscience Unit, Faculty of Psychology, University of Vienna; 5 German Aerospace Center (DLR), Braunschweig, Germany

## Abstract

Airline pilots and similar professions require reliable spatial cognition abilities, such as mental imagery of static and moving three-dimensional objects in space. A well-known task to investigate these skills is the Shepard and Metzler mental rotation task (SMT), which is also frequently used during pre-assessment of pilot candidates. Despite the intuitive relationship between real-life spatial cognition and SMT, several studies have challenged its predictive value. Here we report on a novel instrument interpretation task (IIT) based on a realistic attitude indicator used in modern aircrafts that was designed to bridge the gap between the abstract SMT and a cockpit environment. We investigated 18 professional airline pilots using fMRI. No significant correlation was found between SMT and IIT task accuracies. Contrasting both tasks revealed higher activation in the fusiform gyrus, angular gyrus, and medial precuneus for IIT, whereas SMT elicited significantly stronger activation in pre- and supplementary motor areas, as well as lateral precuneus and superior parietal lobe. Our results show that SMT skills *per se* are not sufficient to predict task accuracy during (close to) real-life instrument interpretation. While there is a substantial overlap of activation across the task conditions, we found that there are important differences between instrument interpretation and non-aviation based mental rotation.

One of the central competences of airline pilots is an extraordinary ability in spatial cognition and mental imagery of static and moving three-dimensional objects in space. A well-known task to investigate these skills is the Shepard-Metzler mental rotation task^1^, which is also frequently used in test batteries for the pre-assessment of pilot candidates. While it has been shown that, e.g., (military) pilots perform better than non-pilots in similar three dimensional tasks^2^, it seems clear that performance in the mental rotation task alone is insufficient for inferring on general real-life spatial cognitions skills. Instead, spatial cognition is a component of the complex concept of situation awareness (SA)^3^,^4^. SA encompasses multiple high-level cognitive processes such as perception, understanding, and projection, which are influenced by and depend on domain- and profession-specific sub-concepts. In aviation, such a sub-concept of SA is spatial situational awareness (SPASA). A decline or break down in SPASA is thus expected to diminish overall SA and consequently can impact flight crew performance. A decline or loss of SPASA, namely, an inability to correctly interpret aircraft attitude, altitude and airspeed in relation to points of reference, can occur in three different stages as recently defined^5^: (1) spatial disorientation that is unrecognized (i.e., unawareness of current state), (2) recognized spatial disorientation which may or may not be identified as such, but with the awareness that the perceived state(s) do not match the current information from instruments or other environmental input (i.e., misinterpretation of current state), and (3) awareness of spatial disorientation including the incapability of reacting to the identified state.

Apparently, a lack of SPASA and the incorrect interpretation of three-dimensional representations of information are well known reasons for accidents, which is why these important skills are tested in pilot assessments. Depending on airline regulations, pilot-assessments include various tests and are carried out by different certified authorities. These evaluations are commonly carried out in a multimodal assessment process which usually tests various cognitive abilities such as mechanical comprehension and technical knowledge, numerical reasoning, short-term memory for auditory and visual information, speed of perception, attention control, reaction time, psychomotor coordination, multiple task capacity, and spatial orientation^6^. To test multiple task coordination the Multiple Task Coordination Test is often applied which requires the simultaneous performance of three different tasks: a compensatory tracking task, an acoustic task and an instrument monitoring task.

In addition to these conceptual considerations, a number of empirical differences between real-life spatial cognition and abstract mental rotation were found. On a behavioral level, differences in mental rotation (i.e., rotating an external object) and egocentric perspective taking (i.e., realigning oneself) have been observed^7^, indicating a difference in the responsible cognitive mechanisms. Furthermore, studies that investigated the relationship between mental rotation and actual spatio-motor performance found such a relationship only in inexperienced or untrained pilots^8^, drivers^9^, and surgeons^10^. Wolf *et al.*^9^, for example, have shown that female novice drivers scored lower in parking skills and a mental rotation paradigm, when compared to a group of males matched for driving experience. However, when repeating the experiment at a later time, this gender difference was only observed for the abstract mental rotation paradigm, yet not for the driving task, where task performance was determined by the level of acquired driving experience, instead.

In combination, these findings raise not only the question if performance in a Shepard-Metzler-like mental rotation task indeed represents the current skill level of real-life spatio-motor performance in a specific profession but also its function as predictor for the future individual development resulting from training and experience. In this study we have designed and implemented an *instrument interpretation* (IIT) task that strongly resembles the attitude indicator, the main instrument used by pilots to determine the attitude of the aircraft and, thus, is close to a pilot’s real-life requirements and affordances while still being highly comparable to the Shepard-Metzler task (SMT) for mental rotation with respect to the presentation of visual stimuli and subject responses by means of pressing one of two buttons (Fig. 1).

However, setting up a comparative study between scenarios that closely resemble real-life, profession-specific requirements versus classic Shepard-Metzler task stimuli impose experimental challenges, particular when using MR neuroimaging methods. First, the form of interaction with the environment could become so complex that it cannot be implemented within the constrained MR scanner environment and requires the use of elaborated simulator models or actual motion-capable simulators, respectively. Even if a realistic implementation was feasible given the constraints of the setup, a task that requires such complex interaction with the environment would induce additional brain activations that limit the comparability with those associated with an abstract mental rotation task (which only requires the subject to observe and press one or two buttons). Second, to suit the experimental constraints of fMRI, real-life scenarios might have to be simplified or distorted to a degree where they no longer pose a valid experimental proxy for the actual phenomenon of interest. The IIT presented herein was specifically designed to combine the basic requirements of the Shepard-Metzler task together with an instrument interpretation referring to the aircraft’s orientation in space.

We investigated the biological substrates recruited for the novel IIT in comparison to the abstract Shepard-Metzler mental rotation task. Further, we compared task performance and task-specific brain activations between both tasks to assess whether the SMT can be regarded a reliable tool to investigate aviation-relevant spatial cognition skills.

## Results

There were no significant behavioural or brain activation differences associated with individual flight experience (i.e., flight hours, aircraft types, and age). On average, participants performed significantly (paired t-test, T = 4.62, p < 0.001) more trials of IIT (mean = 60.33, SD = 16.48) than SMT (mean = 46.50, SD = 12.12) due to faster response times for instrument interpretation (mean = 1.78 s, SD = 0.51s vs. mean = 2.32 s, SD = 0.72 s, paired t-test, T = 3.73, p < 0.002). Task accuracy was significantly lower for IIT (mean = 80.08%, SD = 9.24%) compared to SMT (mean = 88.66%, SD = 6.90%) in a paired t-test (p < 0.005). No significant correlation was found between task accuracies for IIT and SMT (ρ = −0.238, p = 0.341, after outlier removal: ρ = 0.295, p = 0.267). However, a moderate positive correlation was observed for reaction times between mental rotation and instrument interpretation (ρ = 0.559, p = 0.016).

Brain activation, related to both task conditions, was found in visual areas (occipital lobe, fusiform gyrus), superior parietal lobe, dorsolateral PFC, thalamus, putamen, somatosensory cortex, and motor cortex (Fig. 2). Despite this substantial overlap, the contrast between the two conditions revealed task-specific differences. Mental rotation elicited significantly stronger activation in bilateral pre- and supplementary motor areas, and superior and inferior parietal lobe (Fig. 3).

Instrument interpretation, on the contrary, induced significant hyper activation in bilateral fusiform gyri and angular gyri, as well as precuneus and PCC. In the left hemisphere, more activation was found in Broca’s and Wenicke’s area. In the right prefrontal cortex, DLPFC was significantly activated in the IIT > SMT contrast.

## Discussion

The present study investigated the neurobiological correlates of spatial cognition in professional airline pilots while either interpreting information displayed on a realistic attitude indicator (instrument interpretation task, IIT) or performing a generic mental rotation task (Shepard-Metzler task, SMT).

Although a high level of safety has been achieved in aviation due to continuous developments in flight systems and automation, procedures, as well as pilot training, accidents and incidents still occur and human factors such as loss of SA, and specifically, spatial SA, are reported as crucial in accident/incident reports^5^. While the origin of spatial disorientation is often related to physiological processes outside the central nervous system (e.g., in the vestibular system)^11^, overcoming spatial disorientation can be influenced by system and display design. Therefore, most spatial disorientation prevention training programs focus on these physiological processes. More knowledge about the neurological processes could lead to prevention programs also focusing on cognitive training.

A substantial overlap in brain activation related to instrument interpretation and Shepard-Metzler task was found. This implies that similar brain networks are required for abstract mental rotation paradigms and an airplane attitude judgement task that was carefully designed to emulate pilots’ real-life scenarios as closely as possible. The observed task activations for the SMT are consistent with previous studies where similar paradigms were applied^12^,^13^. The predominantly left-lateralized activation in the primary motor cortex can be attributed to the execution of the required button pressing. This interpretation is supported by evidence from a previous study that differentiated these button pressing efforts from spatial cognition processes^14^. Instrument interpretation, when compared to SMT, induced stronger activation in the angular and fusiform gyri. The angular gyrus is central for context-dependent semantic integration of information originating from language-based but also non-language-based sources^15^. Neuronal activity within the fusiform gyrus has been implicated for the recognition of human faces, but also for animate and inanimate objects, in particular for making distinctions of those within a given category^16^,^17^. In accordance with this, a previous study has demonstrated that visual-spatial perspective taking is associated with activation in the (right) fusiform gyrus, when contrasted with a motor representation task, which elicited more activation in the superior parietal lobe^18^. Interestingly, IIT resulted in stronger activation of DLPFC, which might reflect higher engagement of executive functions, including working memory. Notably the latter have been shown to play only a minor role in mental rotation task^19^, thus indicating again the discrepancy between task requirements. The stronger activation in Broca’s and Wernicke’s area during IIT might be linked to verbalization processes that supported the pilots’ perceptual choices.

Most importantly, we found no significant correlation between accuracies of both task types across pilots, which suggests that abstract mental rotation skills *per se* are not sufficient to predict task accuracy during instrument interpretation. Previous studies have shown that abstract mental competences might lead to better outcomes in early training phases of particular spatio-motor tasks. In experienced performers, however, these initial differences were found to mitigate and mainly depend on the respective experience level^9^,^10^. Their findings clearly suggest that spatio-motor expertise within a real-life scenario, such as surgery or car driving, only rely in part on the brain network necessary for abstract mental rotation skills. Differentiating the role of perspective taking versus motor-based mental rotation is also of high relevance for display and instrument design. Maintaining and (re)establishing spatial awareness, i.e., awareness of the current and future aircraft attitude, is a central requirement for pilots.

On the other hand, we observed a significant correlation of reaction times between the two different tasks. Reaction times critically depend on individual performance indicators like vigilance and confidence. Fast processing is, however, not the essential target in the tasks used in our study, and participants were instructed accordingly. In case of airline pilots, correct interpretation of the current state of the aircraft is the basis for appropriate decision making. In general, we did not observe a significant correlation between task accuracies and reaction times. With respect to the SMT, however, there was a significant negative correlation between reaction times and task accuracies, which was not observed for the IIT. This provides additional support that SMT performance is not a reliable predictor for other spatial cognition tasks, such as the IIT and, thus, not a reliable stratification tool in pilot assessment.

Typically, spatial awareness is enabled by the multimodal integration of visual, somatosensory, and vestibular information in the medial superior temporal area^20^. However, the conclusions resulting from this functional processing are usually based on standard, earthbound situations and might be unreliable during flight operations, particular when additionally affected by reduced visibility (e.g., clouds, fog, smoke, low light) and spatial disorientation due to illusions within the human sensory system (e.g., somatogravic or oculogravic illusions)^21^. Therefore, pilots have to routinely supplement their sense of orientation by the use of artificial instruments (i.e., attitude indicators). In current attitude indicators, two different designs can be distinguished: (A) the artificial horizon is moving relative to a fixed illustration of an aircraft (sometimes referred to as inside-out, moving horizon or *western-style* attitude indicators), which was used in this study; (B) a fixed horizon and an aircraft symbol that is moving depending on the bank angle (outside-in or *Russian-style* attitude indicator). Both versions appear to have context- and training-dependent benefits^22^ and there is no clear consensus in the aviation community about the superiority of one or the other. Given that both rely on different modes of perspective taking, a better characterization of the underlying neurobiological systems and their performance in situations with high workload and stress, can be highly informative for future instrument design. Additional experiments can be used to differentiate more clearly the types of mental effort required for the comprehension of a particular display type (e.g., difference between image retention and spatial transformation^19^) to allow for an evidence-based design process that minimizes the cognitive workload or potential ambiguities for the operators.

In summary, while we show that there is a substantial overlap of activation in neural networks across both task conditions, we found that there are also significant differences in activation patterns between instrument interpretation and non-aviation based mental rotation. In combination with the absent correlation between task accuracy for the two conditions, our results indicate that abstract mental rotation skills might not be a reliable predictor for actual real-life spatio-motor skills of pilots and likely other trained professionals. Regarding our experiment design, we show the applicability of aviation related displays as fMRI stimuli. In addition to inducing important differences in brain activation, the novel IIT might be useful in future aviation studies due to the higher face validity compared with abstract tests, such as the SMT.

## Methods

### Study population and fMRI protocol

**Figure 1:**
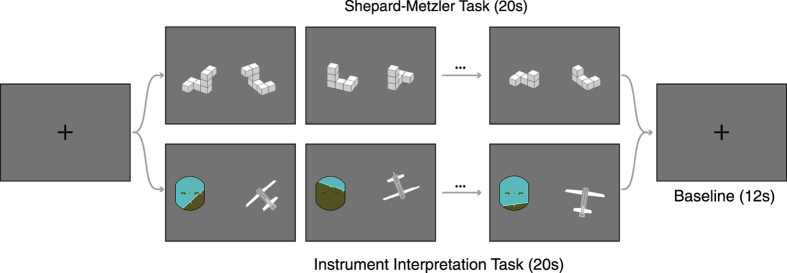
Shepard-Metzler task and three-dimensional instrument interpretation task. Task conditions were presented in alternating blocks (duration 20 s). Subjects had to decide by button press if the left and right images are consistent (i.e., if they are the same object and if the attitude indicator matches the three-dimensional orientation of the airplane with respect to pitch and roll). New stimulus appeared after button press; there was no maximum trial duration. Solutions for the present example sequence are: SMT: correct, incorrect, correct; IIT: incorrect, correct, correct.

**Figure 2:**
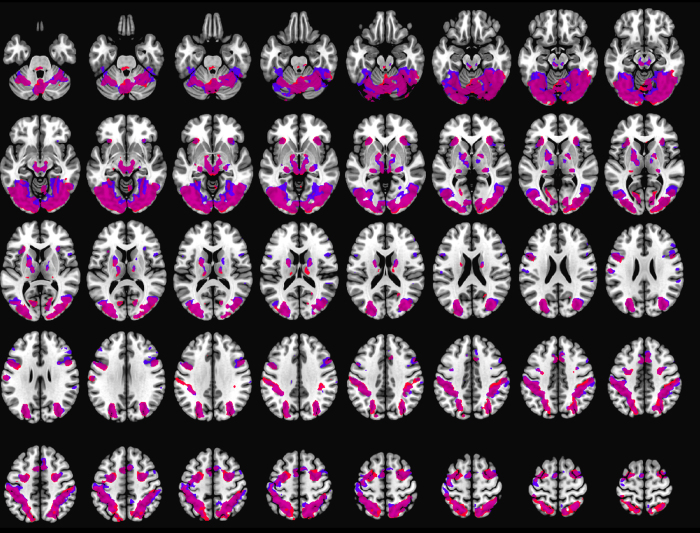
Overlapping brain activation related to Shepard-Metzler task (blue) and instrument interpretation task (red). Overlap of conditions is displayed in purple. Statistical threshold for t-statistics was set to p < 0.05 FWE whole-brain corrected.

**Figure 3:**
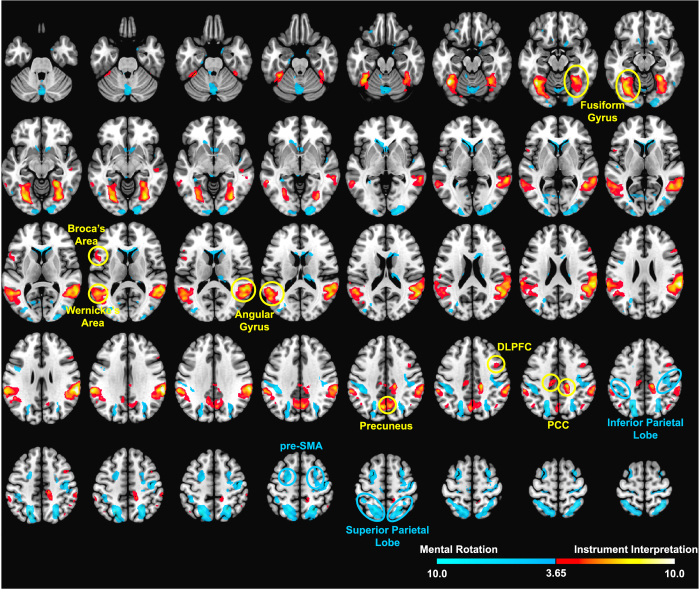
Statistical differences of Shepard-Metzler task (cool) and instrument interpretation task (hot). Statistical threshold for t-statistics was set to p < 0.05 FWE cluster-level corrected. Increased activation during instrument interpretation was found in the fusiform gyri (first row), Broca’s and Wernicke’s area, angular gyri (third row), precuneus, DLPFC, and PCC (fourth row). The Shepard-Metzler task induced more activation in the inferior (fourth row) and superior parietal lobe and the pre-SMA (last row).

We investigated 18 male professional airline pilots (age: 37 ± 7 years, flight hours: 5,500 ± 3,000 h). Subjects performed a Shepard-Metzler mental rotation (SMT) and an instrument interpretation task (IIT) presented in a blocked design (block length = 20 s, 5 repetitions, SMT and IIT alternating) (Fig. 1), while undergoing functional magnetic resonance imaging (fMRI) at the MR Center of Excellence at the Medical University of Vienna. All subjects were financially reimbursed for their participation and provided informed written consent. The study protocol was approved by the institutional review board of the Medical University of Vienna. All methods were carried out in accordance with the approved study protocol, guidelines for good scientific practice, and the Declaration of Helsinki (1964) including current revisions.

Subjects were scanned in a 3 Tesla TIM Trio MR scanner (Siemens Medical, Erlangen, Germany) using the manufacturer’s 32-channel head coil. 270 whole-brain volumes were obtained at a repetition time employing a multiband echo planar imaging (EPI) sequence (TR/TE = 1800/33 ms, resolution = 1.2 × 1.2 × 1.5 mm^3^, matrix size: 128 × 128 × 54 slices).

During the Shepard Metzler mental rotation task (SMT), subjects had to judge whether two three-dimensional objects, which were arbitrarily rotated in space, are identical or different in structure adapted from^1^. For the instrument interpretation task (IIT), a generic attitude indicator and a three-dimensional rendering of a simplified airplane as seen from posterior, were shown. Subjects had to indicate by button press if the pitch and roll displayed on the attitude indicator applies to the airplane (Fig. 1). The fMRI task was implemented in Python (2.7.6) using pyglet (1.1.4, http://www.pyglet.org/) and PyWavefront (0.1.1, https://github.com/greenmoss/PyWavefront) modules. To create the three-dimensional cubes and plane model, Blender (2.68, Stichting Blender Foundation, Amsterdam, Netherlands) 3D animation suite software was used. SMT stimuli were freely rotated along all axes. IIT stimuli were rotated along the principal and secondary axes of the airplane resulting in roll and pitch angles between 2° and 45°. In 20% of all trials, the two stimuli did not match: SMT items were mirrored along the y/z-plane and in the IIT the attitude indicator were left-right flipped, thus resulting in a wrong roll angle (e.g., indicating a 15° roll to the right instead of 15° to the left). There was no time limit for the individual task items, except the overall block length of 20 s.

### Preprocessing and general linear model (GLM) analysis of fMRI data

Data pre-processing and analyses were performed in SPM12b and comprised slice-timing correction^23^, realignment, unwarping, normalization to standard MNI template, and smoothing with a Gaussian kernel (6mm FWHM).

Single-subject GLM analysis comprised two regressors (i.e., mental rotation and instrument interpretation), for which a boxcar function that encoded onset and duration of the respective condition was convolved with SPM’s canonical HRF. The resulting contrast maps were used for subsequent group analysis to localize brain activation associated with the respective task conditions (p < 0.05 whole-brain FWE-corrected) and their potential differences (p < 0.05 cluster-level FWE-corrected, cluster-forming threshold p < 0.001 uncorrected).

## Additional Information

**How to cite this article**: Sladky, R. *et al.* Neurobiological differences in mental rotation and instrument interpretation in airline pilots. *Sci. Rep.* **6**, 28104; doi: 10.1038/srep28104 (2016).
